# Visual effects on tactile texture perception

**DOI:** 10.1038/s41598-023-50596-1

**Published:** 2024-01-05

**Authors:** Roberta D. Roberts, Min Li, Harriet A. Allen

**Affiliations:** 1 School of Psychology, University of Birmingham, Birmingham, B15 2TT UK; 2 School of Psychology, University of Nottingham, Nottingham, NG7 2RD UK

**Keywords:** Sensory processing, Somatosensory system, Visual system, Human behaviour

## Abstract

How does vision affect active touch in judgments of surface roughness? We contrasted direct (combination of visual with tactile sensory information) and indirect (vision alters the processes of active touch) effects of vision on touch. Participants judged which of 2 surfaces was rougher using their index finger to make static contact with gratings of spatial period 1580 and 1620 μm. Simultaneously, they viewed the stimulus under one of five visual conditions: No vision, Filtered vision + touch, Veridical vision + touch (where vision alone yielded roughness discrimination at chance), Congruent vision + touch, Incongruent vision + touch. Results from 32 participants showed roughness discrimination for touch with vision was better than touch alone. The visual benefit for touch was strongest in a filtered (spatially non-informative) vision condition, thus results are interpreted in terms of indirect integration. An indirect effect of vision was further indicated by a finding of visual benefit in some but not all visuo-tactile congruency conditions.

## Introduction

Rougher surfaces tend to be associated with higher friction than smooth surfaces, which facilitates more stable grasp in lifting or moving an object held in precision grip^1^. It is possible to perceive the roughness of a surface by viewing^2,3^ or touching it^4^. However, everyday interactions with surfaces seldom use either sense alone^5–7^. Touched surfaces are typically seen in advance of and during exploratory contact^8^. We ask how surface roughness perception is jointly shaped by the availability of these two sources of sensory input during active touching.

The effect of visual information, presented concurrently with touch, on the perceived roughness of a touched surface may be direct or indirect. An example of a direct effect is when vision provides an estimate of an object’s properties which is combined with the haptic estimate. For example, when estimates of object size in each modality are combined, they are weighted by their reliability, yielding a maximum likelihood estimate (MLE) of the true size of the object^9,10^. The result of such weighted combination is to improve discrimination performance in multisensory relative to unisensory conditions. Bimodal improvements have been found in comparative judgements of object properties, such as size^9,11^, surface slant^12,13^, and judgments of the number of sensory events occurring^14^.

An indirect effect of vision in roughness perception was described by Heller in 1982. In a series of three experiments participants freely explored sandpaper samples using sliding movements of the index finger tip. In two experiments it was shown that accuracy in discriminating the smoothest of 3 different grades of sandpaper was greater for vision and touch compared to vision or touch alone. Heller queried whether the bimodal condition benefit was due to combining visual and haptic information or whether vision provided information about the manner of movement which assisted haptic processing. To address this contrast, equivalent to the direct vs indirect use of vision in the terminology introduced above, Heller ran a third experiment. In that experiment an additional bimodal condition was introduced in which participants felt the sandpaper while viewing the finger moving over the surface, however, the surface details of the sandpaper were obscured using a visual filter. Performance in the two bimodal conditions was found to be the same. Heller concluded that the bimodal benefit arose from vision improving the control of movements used to explore the sandpaper. That is, an indirect rather than a direct benefit of vision. In other studies of roughness perception, bimodal conditions have been shown to leave performance unchanged^15^ or lead to performance corresponding to the average of the performance in each modality alone^7,16^. The latter has been attributed to an equal division of attention across touch and vision^16^. In such cases, we suggest that vision may also be thought of as acting indirectly on tactile perception.

The present experiment examined whether there are bimodal improvements in roughness discrimination of square wave gratings in the 1600 μm (1.6 mm) spatial period range. Performance under bimodal touch and vision (including normal vision, vision with surface details obscured, congruent or incongruent vision) was compared with touch only performance. In particular we examined whether roughness discrimination improvements or impairments were attributable to the multisensory integration of visual and tactile roughness cues or could be accounted for by an indirect effect of vision on touch such as changing tactile spatial acuity^17^. In the event that visual effects on performance also arise from a direct route, it might be expected that performance would be dominated by highly salient visual cues to roughness. Therefore, we also examined whether apparent visual roughness directly affects roughness discrimination under conditions of suprathreshold visual roughness difference cues.

Together, these questions were addressed by comparing roughness discrimination using touch alone with conditions in which visual cues (i) were veridical compared with vision that was blurred using a filter and (ii) afforded suprathreshold discrimination but differed from touch in a manner congruent, or incongruent, with the correct response. If vision impacts directly on bimodal roughness perception we would expect bimodal roughness discrimination to be improved with veridical and congruent vision relative to touch alone. If the effects of vision are indirect, performance should be enhanced with any visual input.

In touch, surface roughness may be determined using spatial or vibrational cues^18^ mediated by slowly (SA) or rapidly (RA) adapting afferents. Fine textures (spatial frequency < 200 microns) are mediated primarily by the vibrational channel, which requires sliding contact between the surface and the skin to elicit vibrations. In contrast, coarser textures are primarily mediated by the spatial channel for which a static pressing contact is sufficient for the necessary transfer of surface topography onto the skin. A tDCS study of Yau et al.^19^ has shown that stimulation over visual cortex affects tactile perception of spatial orientation whereas stimulation over auditory cortex affects tactile perception of vibration frequency. This suggests that static touch might offer the better prospect for demonstrating interactions between touch and visual modalities. Therefore, in contrast to previous studies of the integration of touch with vision which have generally allowed free exploration including sliding touch, the present study focuses on static, pressing touch.

## Materials and methods

### Apparatus

**Figure 1:**
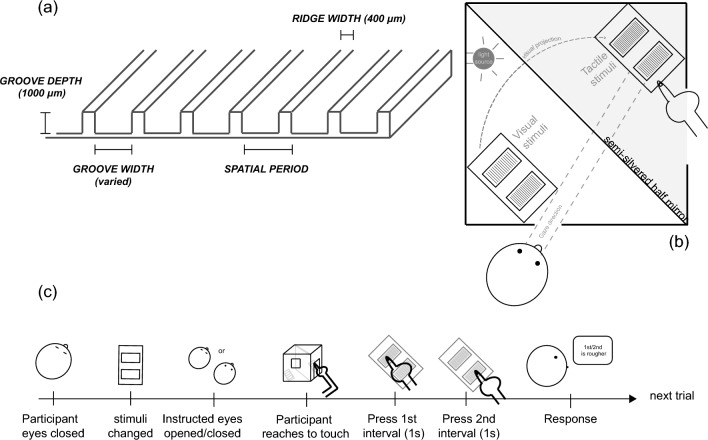
Experimental set up and sequence.

The stimuli comprised 6 rectangular blocks of Tufset polyurethane (35 × 29.5 × 6 mm). Computer numerical control methods were used to cut square-wave patterns into one surface (35 × 29.5 mm) of each block (Fig. 1a). Blocks with spatial periods of 1580 and 1620 μm functioned as tactile stimuli. Pilot testing established that this difference produced correct roughness discrimination ~ 75% of the time using touch. The 4 remaining blocks were used to project reflected images onto the locations of the tactile stimuli. These visual stimuli had periods of 1360, 1580, 1620 and 1920 μm. The ridge widths of all stimuli were 400 μm. A separate test established vision-alone discrimination performance with the stimuli. Discrimination was at chance for 1580 versus 1620 μm gratings whereas both  the 1580 and 1620 gratings were fully discriminable from those with 1360 and 1920 μm spatial periods.

Stimuli were presented using a mirror-box setup similar to that described by Yanagisawa and Takatsuji^20^, comprising a box with dimensions 29 × 31 × 22 cm, (H × W × D), divided at 45 degrees by a semi-silvered mirror. The pair of tactile and a pair of visual stimuli were placed on opposite sides of, and equidistant from, the mirror. Visual stimuli were located on the reflecting side of the mirror, their image therefore projected onto the perceived location of the tactile stimuli. A pair of apertures in the mirror-box allowed participants to view projected visual stimuli and to touch tactile stimuli. Two further apertures, unseen by participants, allowed the experimenter to change the stimuli. Visual stimuli were lit by a light source placed 32 cm above them. Luminance levels were manually adjusted to ensure that observers were able to see a weak image of their hand and finger making contact with the touch stimuli while observing the visual stimuli very clearly (Fig. 1b).

### Participants

Participants comprised 33 University of Birmingham graduate and undergraduate students (17 females) aged between 18 and 44 (mean = 25.3 ± 5.2) years. Each participant was paid £7 per hour for taking part. Participants were naïve to the aims of the study. All participants reported normal or corrected-to-normal vision and no sensory impairments of, or injuries to, the hands. All participants gave written informed consent before taking part. The experiments were conducted according to the protocol approved by the Science, Technology, Engineering and Mathematics Ethical Review Committee of the University of Birmingham (ERN_09-528AP24).

### Experimental design

Every participant was tested on a sequence of 140 trials. Each trial belonged to one of 7 experimental conditions: 3 visuo-tactile baseline conditions and 4 visuo-tactile congruency conditions. Trials from the different conditions were randomly interleaved. Thus congruency trials were randomly interleaved with baseline trials. The presentation order of standard and comparison stimuli on a trial was counterbalanced. The trial and stimulus order was determined in advance for each participant.

### Visuo-tactile baseline trials (touch only, veridical and filtered vision conditions)

Participants touched the two tactile stimuli in each trial. The pair of gratings were touched in succession (two temporal intervals). Participants reported which interval contained the rougher grating. Tactile stimuli were presented with and without visual input. In the touch only (T) condition participants kept their eyes closed throughout the trial. In eyes open conditions visual stimuli, reflected onto the location of the tactile stimuli, provided either veridical information about tactile spatial period (VVT), or no information about tactile spatial period due to occlusion from a blurring plastic filter (FVT).

### Visuo-tactile congruency trials (congruency conditions)

In the 4 congruency conditions we manipulated the bimodal congruency between tactile and visual cues. In one stimulus interval, (standard), the paired visual and tactile stimuli were identical in spatial period. In the other stimulus interval, (comparison), spatial period differed between the modalities. The direction of this difference, relative to the standard stimulus, formed the congruency contrast. When the two modalities differed in the same direction, relative to their modality equivalents in the standard interval, they were classed as congruent (CVT), e.g. both visual and tactile stimuli in the comparison interval were higher/lower than those in the standard interval. When the spatial period of a standard stimulus was between that of the visual and tactile comparison stimuli, the trial was classed as incongruent (IVT). For half of the trials the coarser tactile stimulus (1620 μm) was used in the comparison interval. The finer of the tactile pair (1580 μm) was used in the other half. Table 1 shows stimulus combinations. Visual stimuli in congruency trials were fully discriminable from each other.

**Table 1:** Experimental conditions in the bimodal roughness discrimination task.

Conditions	Spatial period (μm)			
	Standard interval		Comparison interval	
	Tactile stimuli	Visual stimuli	Tactile stimuli	Visual stimuli
Visuo-tactile baseline				
Touch (T)	***1580*** (1620)	–	***1620*** (1580)	–
Filtered-vision + touch (FVT)	***1580*** (1620)	blurred	***1620*** (1580)	blurred
Veridical-vision + touch (VVT)	***1580*** (1620)	***1580*** (1620)	***1620*** (1580)	***1620*** (1580)
Visuo-tactile congruency				
Congruent-vision + touch (CVT)	***1580*** (1620)	***1580*** (1620)	***1620*** (1580)	***1920*** (1360)
Incongruent-vision + touch (IVT)	***1580*** (1620)	***1580*** (1620)	***1620*** (1580)	***1360*** (1920)

The spatial period of the different stimulus combinations is shown in microns. Each row represents the stimuli presented on a trial. Visual and tactile stimuli presented together are shown in neighbouring columns and in the same font. Bold, italicised values represent spatial periods of stimuli presented within the same trial. Bracketed values in normal font represent stimuli presented together on stimulus counter-balanced trials. Note in the congruency conditions: either (in bold) the coarser tactile stimulus was modified (1620 + V1920 or V1360) in the comparison interval or (in parentheses) the finer tactile stimulus was modified (1580 + V1920 or V1360) in the comparison interval. The presentation order of the standard and comparison interval were counterbalanced.

### Vision only trials

Eight naïve participants (mean age: 26 ± 2.7 years; 5 female) were asked to make visual discriminations of the test stimuli used in the visuo-tactile baseline and congruency trials.

### Experimental protocol

Before testing, participants used soap and water to remove any dirt particles or grease from their hands. They then sat at a table on which the mirror-box was placed (Fig. 1b). The position of the mirror-box was adjusted to a comfortable height, allowing participants to easily reach into the box while viewing the visual stimuli. The viewing distance to the visual stimuli was approximately 29 cm (visual angle 5.6°). Participants closed their eyes between trials. During this eyes-closed period the experimenter reached into the mirror-box and placed the tactile and visual stimuli in the appropriate positions, ensuring reflection of the visual simuli onto the location of the tactile stimuli. Once the stimuli were in place, the experimenter informed the participant whether to keep their eyes closed (touch condition) or open their eyes and look at the position to which they would reach (all other conditions). Following a ‘go’ prompt, participants in eyes-open conditions, opened their eyes, directed their gaze towards the perceived location of the tactile stimuli and reached into the tactile-aperture to touch the two tactile stimuli in succession. In the touch condition (when eyes remained closed), the experimenter assisted participants with hand positioning. In all conditions, the participants were instructed to use a pressing contact lasting approximately 1 s. At the end of contact, participants reported which interval contained the rougher surface (Fig. 1c). Compliance with instructions was visually monitored by the experimenter. Over-ear headphones, playing white noise, were used to mask any auditory roughness cues. The experimenter, positioned behind the mirror-box, manually changed the stimuli on each trial.

The entire experiment lasted approximately 80 min with a practice session at the start, and short breaks halfway through and at participants’ request.

### Data analysis

The proportion of correct trials were calculated for each condition. Discrimination performance for each participant was characterised using a visual benefit measure, calculated by subtracting the proportion of correct trials in the touch only condition from each visuo-tactile score. The data of two participants were excluded from further analyses. In one case the participant used sliding rather than pressing touches. In the other case experimental results were more than 2 standard deviations away from the group means in 3/7 conditions. For the remaining 31 participants, 5/217 individual data points, exceeding 2 SD of the condition mean, were replaced using stochastic regression imputation method in SPSS^21^.

An alpha of 0.05 was adopted for statistical analyses. One-tailed t-tests are reported in anticipation of the visual benefit reported by Heller^5^.

## Results

### Visuo-tactile baseline data

**Figure 2:**
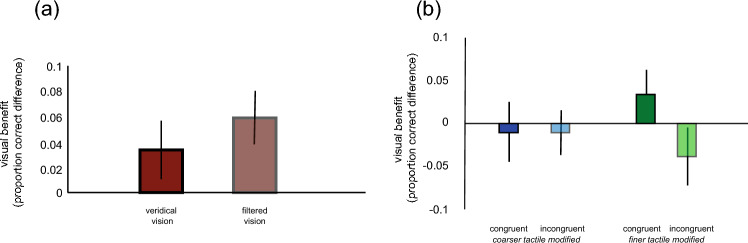
Bar graph showing the mean visual benefit (vision + touch − touch alone) in the different vision conditions. The visual benefit of combining touch with veridical or filtered vision is shown in (**A**) on the left. The visual benefit of combining touch with congruenct or incongruent vision is shown in (**B**) on the right. 1 SE of the mean is shown.

The overall mean proportion of correct responses was 0.68 (SD = 0.11) in the touch condition (T), 0.71 (SD = 0.14) for the veridical vision bimodal (VVT) condition and 0.74 (SD = 0.13) for the filtered vision bimodal (FVT) conditions. The visual benefit of veridical and filtered visual information over touch alone are shown in Fig. 2A.

This baseline data was examined using one sample t-tests to compare the visual benefit in the two bimodal conditions with zero. While the visual benefit of veridical visual information was not reliable t(30) = 1.425, *p* = 0.082, there was a significant benefit to having filtered visual information, t(30) = 2.834, *p* = 0.004. The difference between the veridical and filtered vision benefit was not significant, t(30) = − 0.859, *p* = 0.199.

### Congruency data

The proportion of correct responses when the coarser tactile stimulus was paired with (i) congruent visual stimuli was 0.67 (SD = 0.18) and (ii) incongruent visual stimuli was 0.65 (SD = 0.15). When the finer tactile stimulus was paired with visually congruent surfaces the proportion of correct responses was (i) 0.70 (SD = 0.15) but lower at (ii) 0.65 (SD = 0.16) when paired with visually incongruent stimuli.

Congruency effects on the visual benefit data (see Fig. 2B), were examined using a repeated-measures ANOVA with crossmodal congruency (congruent or incongruent) and visually-modified tactile stimulus (coarser or finer) as within-subject factors. There were no main effects of congruency, F(1,30) = 0.161, *p* = 0.691, or visually-modified tactile stimulus, F(1.30) = 1.003, *p* = 0.325. There was a significant interaction between the two factors, F(1,30) = 4.853, *p* = 0.035 driven by a significant congruency effect (congruent–incongruent) for the finer tactile stimulus, t(30) = 1.770, *p* = 0.043 but no effect for coarser tactile stimuli, t(30) = 0.033, *p* = 0.487.

### Vision only data

In the 8 participants tested, visual discrimination of the 1580 versus 1620 μm gratings were at chance for the veridical, 0.48 (SD = 0.10), and filtered, 0.52 (SD = 0.02), vision conditions. Roughness discrimination of the visual stimuli used in the congruent conditions was at or near ceiling level of 0.95 or above.

## Discussion

Tactile cues to roughness can be spatial or vibratory and are associated with static or sliding contact respectively. With coarser textures, whose features are of the order of a thousand microns, static contact is sufficient to discriminate roughness^22,23^. In this study we asked whether, with static contact, vision would enhance sensitivity to roughness. The answer to this question was a qualified yes. Visual benefit to tactile discrimination was most clearly evident in the presence of visual cues lacking information about surface roughness. However, vision benefits were also, under certain conditions, evident with suprathreshold visual roughness cues.

In some ways the present findings are similar to those reported by Heller^5^. Both sets of studies report bimodal improvement over touch alone in the presence of non-informative (filtered) visual inputs. While Heller reported no difference between spatially informative (veridical) and filtered vision in his bimodal conditions the present results suggest a gradient of roughness sensitivity between touch alone, and touch plus spatially informative and spatially non-informative vision. A major difference between the studies is that, here, the contact was static, whereas in Heller’s case sliding was permitted. Heller proposed that visual guidance of exploratory motion underlay his findings of a bimodal benefit to roughness discrimination in the presence of vision. In the present case of a simple pressing contact this explanation is unlikely. Our first main finding, that some benefit occurs whether or not the visual detail is available, suggests that part of the effect arises from vision directed toward and maintained at the touched location.

In our experiment, the active finger can be seen making contact with the ‘apparent’ surface texture. Obscured visual inputs can facilitate the processing of tactile stimuli through attentional mechanisms, provided the stimulated body part is seen^24,25^. It is possible that our participants may have benefitted from vision through its attention related enhancement of tactile processing. Seeing the touched skin can also affect low-level mechanisms in the somatosensory cortex, sharpening the receptive fields of SA1 afferents^26^ whose activity underlies tactile spatial acuity^27^. In our experiment, the static contact conditions meant successful discrimination was likely reliant on SA1 signals, making receptive field refinement a candidate mechanism for the visual benefit we observed.

A second major finding in our study is that there is sometimes a benefit from congruent relative to incongruent visual stimuli. In full vision conditions it is necessary to look at and reach towards the tactile stimuli to feel and judge their relative roughness. We suggest in this phase of a trial expectations of the roughness of specific stimuli are created. When the finger subsequently makes contact, discrepancies between expected and actual roughness signals would be apparent in both congruent and incongruent conditions leading to shifts in perception towards or away from the expected roughness.

In relation to our first finding, the results of our vision only experiment suggest that vision in the filtered and veridical visuo-tactile conditions was likely to provide provided little to no direct information about the spatial features of the touched surfaces. However, it is worth noting that we did not measure visual discrimination for the individual participants in the visuo-tactile experiment. This group may have been more varied in their visual discriminative abilities for veridical vision than the results of the vision only experiment suggest. In relation to our second finding, the congruency conditions were designed to test the effects of stronger visual signals of roughness difference on tactile roughness discrimination. Under such conditions, direct effects of vision on touch would predict improved roughness discrimination with congruent signals and impaired discrimination with incongruent visuo-tactile stimuli. With congruent stimuli the (direct) visual benefit might have been expected to be additional to that seen with filtered vision. In contrast, our predicted indirect effect of vision on touch would have been evident as a uniform visual benefit across all bimodal conditions. Alternatively, in the presence of strong visual cues to roughness difference participants could have responded solely to visual cues producing ceiling performance in congruent conditions and no correct answers in incongruent conditions. None of these outcomes were observed here. Rather there was an interaction in which visuo-tactile congruency had no effect on visual benefit when the standard was 1620 μm but showed a benefit when the standard was 1580 μm. While these data are hard to account for straightforwardly, they do indicate that visual roughness cues are processed, but are not directly combined with tactile cues and can, under certain conditions, lead to loss of the visual benefit of non-informative vision.

Previous investigations of touch-visual interactions in roughness discrimination have left tactual explorations unconstrained or restricted contact to sliding conditions^16,28^ thus allowing both spatial and vibration haptic cues. Here we have studied roughness perception in pressing, a mode of contact most relevant to reach and grasp. Vision may be especially important just prior to contact, not only guiding reaching but setting up expectations about likely contact conditions. These expectations might include what the object will feel like and how to control contact forces to ensure stable grasp or optimise stimulation of relevant sensory receptors for discrimination. In our previous work we have found evidence of a role for force control in roughness discrimination^29^. In future research it will be important to examine effects of vision on contact forces and exploratory dynamics in the assessment of surface roughness through touch.

## Data Availability

The datasets generated and analysed during the current study are available in the OSF repository, https://osf.io/6fyp7/?view_only=d15cad264be8418cbd49017ddd3f7cdd.
